# Effectiveness and Feasibility of Telehealth-Based Dietary Interventions Targeting Cardiovascular Disease Risk Factors: Systematic Review and Meta-Analysis

**DOI:** 10.2196/49178

**Published:** 2024-02-16

**Authors:** Rupal Trivedi, Shaimaa Elshafie, Randall Tackett, Henry Young, Elisabeth Lilian Pia Sattler

**Affiliations:** 1 Department of Clinical and Administrative Pharmacy College of Pharmacy University of Georgia Athens, GA United States; 2 Department of Nutritional Sciences College of Family and Consumer Sciences University of Georgia Athens, GA United States

**Keywords:** telehealth, dietary interventions, cardiovascular diseases, risk factors, self-management, systematic review and meta-analysis

## Abstract

**Background:**

Telehealth-based dietary interventions were recommended for cardiovascular disease (CVD) management during the COVID-19 pandemic; however, data regarding their effectiveness and feasibility are limited.

**Objective:**

We aimed to examine (1) the effectiveness of telehealth-based dietary interventions in improving clinical CVD risk factors and (2) the feasibility of these interventions among individuals with CVD.

**Methods:**

To conduct this systematic review and meta-analysis of randomized controlled trials (RCTs), 2 investigators searched PubMed, Cochrane Library, Web of Science, and ClinicalTrials.gov databases based on predetermined search terms and included English-language RCTs published between January 2000 and July 2022. The Cochrane Risk of Bias tool was used to assess RCT quality. To evaluate intervention effectiveness, weight, BMI, systolic and diastolic blood pressure, and levels of total cholesterol, low-density lipoprotein cholesterol, high-density lipoprotein cholesterol, triglycerides, or blood glucose were compared postintervention in telehealth and usual care (UC) groups. Feasibility was determined through the number of participants retained in intervention and UC groups. Pooled data for each CVD outcome were analyzed using a random effects model. Mean difference (MD), standardized MD, or risk ratio were calculated using R software.

**Results:**

A total of 13 RCTs with 3013 participants were included in the analysis to assess the effectiveness and feasibility of telehealth-based dietary interventions among individuals with CVD. Participants had a mean age of 61.0 (SD 3.7) years, and 18.5% (n=559) were women. Approximately one-third of RCTs were conducted in the United States (n=4, 31%). Included studies used telephone, app, text, audio-visual media, or website-based interventions. Of the 13 included studies, 3 were of high quality, 9 were of moderate quality, and only 1 was of low quality. Pooled estimates showed systolic blood pressure (MD –2.74, 95% CI –4.93 to –0.56) and low-density lipoprotein cholesterol (standardized MD –0.11, 95% CI –0.19 to –0.03) to be significantly improved among individuals with CVD as a result of telehealth-based dietary interventions compared to UC. No significant difference in effectiveness was detected for weight, BMI, and levels of diastolic blood pressure, total cholesterol, high-density lipoprotein, and triglycerides between telehealth-based dietary interventions and UC among those with CVD. There was no significant difference between the feasibility of telehealth-based dietary interventions versus UC. Significant *I*^2^ indicated moderate to considerable heterogeneity.

**Conclusions:**

Telehealth-based dietary interventions show promise in addressing CVD risk factors.

## Introduction

Cardiovascular diseases (CVDs) impact 126.9 million American adults and are associated with 5.0 million annual hospitalizations, 874,613 annual deaths, and US $378 billion in medical expenditures in the United States [1]. CVD diagnoses are adversely impacted by lifestyle-related CVD risk factors [2]. About 80% of CVD risk in the United States can be attributed to lifestyle factors, including the consumption of an unhealthy diet, smoking, physical inactivity, alcohol use, obesity, uncontrolled levels of blood pressure, total cholesterol, and blood glucose [2]. Out of these lifestyle-related factors, the consumption of an unhealthy diet contributes to the largest proportion of CVD risk among Americans [2].

For primary and secondary CVD prevention, clinical guidelines endorse the maintenance of a healthy body weight, engagement in physical activity, smoking cessation, limited alcohol consumption, and a healthy diet [3-6]. Registered dietitian nutritionist (RDN)-led medical nutrition therapy has been shown to successfully improve dietary consumption among individuals with CVD [3,5,6]; however, limited access to health care services due to long travel distances and lack of adequate transportation to health care facilities presents significant barriers to obtaining effective medical nutrition therapy, particularly in rural communities [7-11].

Telehealth technologies provide a solution for overcoming these barriers by administering and supporting clinical health care over a long distance [12,13]. Through the use of the internet, videoconferencing, streaming media, electronic health records, and terrestrial and wireless communication tools, a variety of medical conditions have been successfully managed, including CVD [14,15]. Furthermore, the American Society for Preventative Cardiology recommended telehealth technologies for the dissemination of RDN-led medical nutrition therapy among those diagnosed with CVD during the COVID-19 pandemic [16]. While RDN-led telehealth-based medical nutrition therapy showed great promise in expanding patient care infrastructure during the pandemic, data regarding the effectiveness and feasibility of telehealth services among individuals with CVD are limited [16]. Previous research used small sample sizes, lacked inclusion of individuals with CVD as the target population, and did not assess telehealth-based dietary interventions [17]. Therefore, the objective of this study was to determine the effectiveness and feasibility of telehealth-based dietary interventions among individuals with CVD.

## Methods

### Study Design

We conducted a systematic review and meta-analysis, guided by the PRISMA (Preferred Reporting Items for Systematic Reviews and Meta-Analyses) 2020 [18].

### Inclusion Criteria

Randomized controlled trials (RCTs) published in the English language between January 2000 and July 31, 2022, that compared telehealth-based dietary interventions and usual care (UC) among adults aged ≥18 years with CVD were included in this study. Eligible RCTs involved participants with CVDs, including coronary heart disease, heart failure, hypertension, individuals with CVD with a history of cardiac events (myocardial infarction, acute coronary syndromes, angina, and revascularization), and individuals with CVD with a history of cardiac procedures (percutaneous coronary intervention and coronary artery bypass graft surgery) [19,20]. Interventions were considered as telehealth-based dietary interventions if diet-related information was conveyed through synchronous or asynchronous points-of-contacts between a patient and a health care professional via medical education systems or information and communications technologies, such as telephones, cellular phones, web-based systems, and video technologies [21,22]. Participants in UC groups received some form of in-person nutritional care as a part of CVD management. If the involvement of nutritional care in UC was not clearly stated in the RCTs’ papers, authors R Trivedi and ELPS contacted the study authors to gain clarity. In the case where authors did not reply with clarification, the corresponding RCT was excluded from this systematic review and meta-analysis. To determine the effectiveness of telehealth-based dietary interventions, clinical CVD risk factors (weight, BMI, systolic and diastolic blood pressures, and levels of total cholesterol, low-density lipoprotein [LDL] cholesterol, high-density lipoprotein [HDL] cholesterol, triglycerides, or blood glucose) were assessed in postintervention in telehealth, and compared to UC groups. To determine patient feasibility toward the telehealth-based dietary interventions, the included studies reported the number of participants retained by the RCT in both intervention and UC groups. 

### Search Strategy

PubMed, Cochrane Library, Web of Science, and ClinicalTrials.gov databases were searched using the following key terms and Medical Subject Headings terms: (“diet” or “nutrition” or “nutrition status” or “status, nutrition” or “nutritional science” or “science, nutritional” or “nutrition science” or “science, nutrition”) and (“telehealth” or “telemedicine” or “mobile health” or “health, mobile” or “mhealth” or “telehealth” or “ehealth”) and (“cardiovascular disease” or “cardiovascular diseases” or “disease, cardiovascular” or “diseases, cardiovascular” or “heart disease” or “cardiac disease” or “cardiac disorder” or “heart disorder” or “vascular disease” or “disease, vascular”) and (“rand*”). An experienced librarian reviewed and confirmed this search strategy (Multimedia Appendix 1).

Using the predetermined search string, 2 investigators (R Trivedi and SE) first screened titles and abstracts to determine eligibility independently. Further scrutiny was given to the full text of the selected papers to determine whether eligibility was sustained. The included and excluded records were compared between the 2 investigators, and no unresolved disagreements needed to be reconciled through the input of a third investigator (ELPS).

### Data Extraction

Study characteristics manually extracted from each RCT included National Clinical Trial identifier number, first author’s last name, year of publication, cardiovascular condition, interventions and their duration, follow-up durations after intervention initiation, country in which the study took place, location type (urban or rural), and total number of randomized participants. Participant characteristics extracted from each RCT included age, gender, race or ethnicity, and education and income levels. Continuous clinical outcome data included postintervention weight (kg or lbs), BMI (kg/m^2^), systolic and diastolic blood pressure (mmHg), and levels of total cholesterol (mg/dL or mmol/L), LDL cholesterol (mg/dL or mmol/L), HDL cholesterol (mg/dL or mmol/L), triglycerides (mg/dL or mmol/L), or blood glucose (mg/dL or mmol/L) from intervention and UC groups, respectively. The dichotomous feasibility outcome data included the number of participants retained in intervention and UC groups [23-28].

### Risk of Bias

Study quality was determined independently by R Trivedi and SE through the Cochrane risk of bias tool, assessing randomization, allocation concealment, blinding of participants and personnel, blinding of outcome assessment, incomplete or selective reporting, and external sources of bias [29]. The decision on the RCTs’ overall quality was based on the number of unmet or insufficiently described criteria and the following thresholds: more than 3 (low quality), 2-3 (moderate quality), and less than 2 (high quality). All conflicts were resolved through discussion by the 2 investigators, and no further input was required by ELPS as a third investigator.

### Analytic Plan

Pooled data for each outcome was assessed using a random effects model. Data for continuous variables were assessed either as weighted mean difference (MD) or standardized mean difference (SMD) using the inverse-variance model while our dichotomous variable was assessed as a risk ratio using the Mantel-Haenszel (DerSimonian-Laird method) model to account for the variations among studies. An MD was calculated when outcomes were reported in a uniform measurement scale and SMD was calculated when nonuniform measurement scales were used. Since participants from the included studies were assigned to intervention and UC groups at random, baseline characteristics of participants between groups of each study were assumed to be similar. Therefore, no analysis was conducted to detect differences between the intervention and the UC groups at baseline. Visual inspection of the forest plots and the statistically measured *I*^2^ determined the heterogeneity among the analyzed studies. The level of heterogeneity was based on the following *I*^2^ thresholds: up to 29.99% (low), 30%-59.99% (moderate), 60%-74.99% (substantial), and 75%-100% (considerable). A sensitivity analysis was performed if significant heterogeneity was detected through analysis and the outcome involved more than 7 RCTs, ensuring that the *I*^2^ estimate was reliable and not overestimated by a small number of studies analyzed [30]. The sensitivity analysis was performed based on the type of telehealth intervention and the follow-up duration after intervention initiation. Further assessment to determine the source of heterogeneity included influence analysis. Influence analysis, or “leave-one-out” approach, assesses between-study heterogeneity by quantifying the influence of any one RCT on the overall summary estimate that is calculated in a meta-analysis, which is beneficial in identifying potential sources of error or bias introduced by an RCT [31-33]. Visual inspection of the funnel plot and the statistical Egger test were used to assess publication bias for outcomes with more than 10 RCTs to ensure adequate power for the test [29]. All analyses were conducted using the *meta* and *metafor* packages in R (version 4.2.2; R Foundation for Statistical Computing) with a *P*≤.05 (95% CI) set as a statistically significant level.

## Results

### Literature Search Results

**Figure 1:**
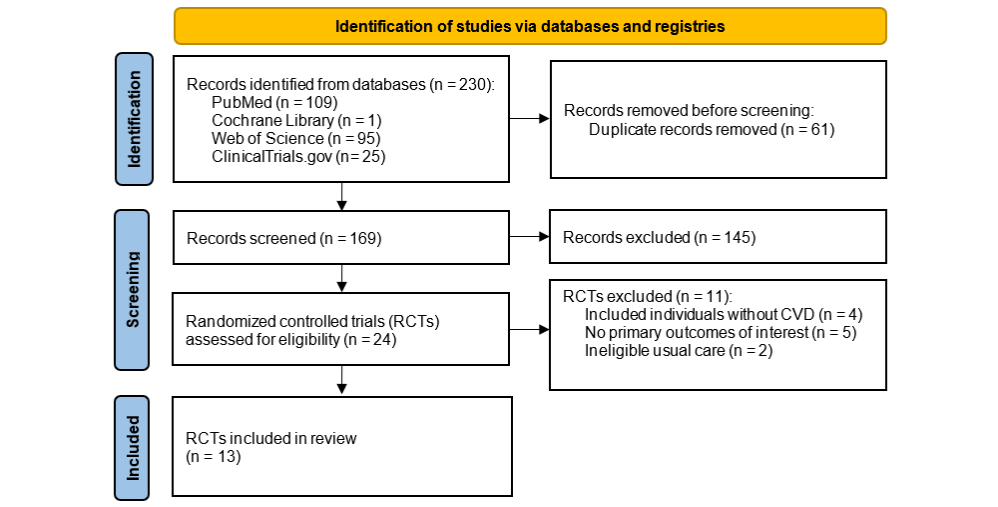
PRISMA flow diagram of screened and selected studies.

Our search string initially returned 230 records from PubMed, Cochrane Library, Web of Science, and ClinicalTrials.gov databases. Out of the 230 records, 61 were duplicates and the remaining 169 records’ titles and abstracts were initially screened. After applying the inclusion and exclusion criteria, 145 records were excluded from this study. A total of 24 RCTs’ reports were further assessed for eligibility, out of which 11 RCTs were excluded in secondary screening. Of these excluded RCTs, 4 included non-CVD participants, 5 did not report any primary outcomes of interest, and 2 did not incorporate an eligible UC. Based on the screening completed, a total of 13 RCTs were included in this study (Figure 1).

### Study Characteristics

A summary of the study and participant characteristics of the included 13 RCTs [34-46] are shown in Multimedia Appendix 2 [34-46]. A total of 3013 participants were analyzed in this study. The mean age was 61.0 (SD 3.7) years and 18.5% (n=559) were women. Cardiovascular conditions in the included studies were a “general CVD diagnosis” (n=3), hypertension (n=3), individuals with CVD who experienced cardiac events (ie, myocardial infarction, acute coronary syndromes, angina, and revascularization, n=3), individuals with CVD who underwent cardiac procedures (ie, percutaneous coronary intervention and coronary artery bypass graft surgery, n=2), heart failure (n=1), and coronary heart disease (n=1). The trial locations included United States (n=4), Europe (n=4: Netherlands, United Kingdom, Spain, and Sweden), New Zealand (n=2), Australia (n=1), Korea (n=1), and Japan (n=1). Except for 1 trial [38], all RCTs took place in an urban setting. Intervention durations ranged from 4 weeks to 48 weeks. Follow-up durations after intervention initiation varied from 4 weeks (n=1), 6 weeks (n=1), 8 weeks (n=1), 12 weeks (n=2), 16 weeks (n=1), 24 weeks (n=4), 40 weeks (n=1), 48 weeks (n=1), to 48-56 weeks (n=1). The types of telehealth-based dietary interventions were based on apps (n=7), text messaging (n=3), telephone calls (n=1), web interaction (n=1), and an audio-visual media device (n=1). Of note, few RCTs included in this study involved RDNs in the dissemination of the telehealth-based dietary interventions (n=4) and UC (n=1).

### Risk of Bias

The quality of the included RCTs, assessed based on the Cochrane risk of bias tool [29], is presented in Table 1. Out of the 13 included studies, 3 were assessed to be of high overall quality, while 9 were of moderate quality, and only 1 was of low quality. The majority of RCTs did not mask their participants; however, the absence of masking outcome assessors and unclear description of allocation concealment additionally contributed to the moderate and low assessments of overall quality.

**Table 1:** Overall quality assessment of included randomized controlled trials based on the Cochrane risk of bias tool.

Study	Random sequence generation	Allocation concealment	Masking of participants	Masking of outcome assessors	Avoided incomplete or selective reporting	Free from other external sources of bias	Overall quality
Friedberg et al [34]	++^a^	++	–^b^	++	++	++	High
Chow et al [35]	++	++	–	++	++	++	High
Dale et al [36]	++	++	–	–	++	++	Moderate
Eyles et al [37]	++	++	–	–	++	++	Moderate
Barnason et al [38]	++	+^c^	–	–	++	++	Moderate
Choi et al [39]	++	+	–	–	++	–	Low
Engelen et al [40]	++	–	–	–	++	++	Moderate
Dorsch et al [41]	++	+	–	–	++	++	Moderate
Riches et al [42]	++	++	–	–	++	++	Moderate
Bae et al [43]	++	++	–	++	++	++	High
Peydró et al [44]	++	++	–	++	++	–	Moderate
Michelsen et al [45]	++	++	–	–	++	++	Moderate
Nagatomi et al [46]	++	+	–	–	++	++	Moderate

^a^Low risk of bias.

^b^High risk of bias.

^c^Moderate risk of bias.

### Meta-Analysis

**Figure 2:**
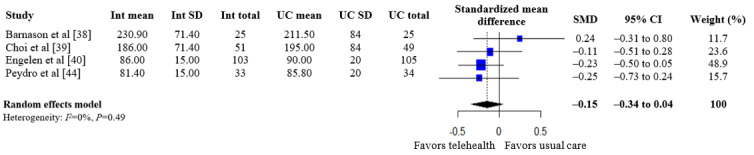
Forest plot of standardized mean differences in weight for telehealth-based dietary intervention (Int) and usual care (UC) groups [38-40,44].

**Figure 3:**
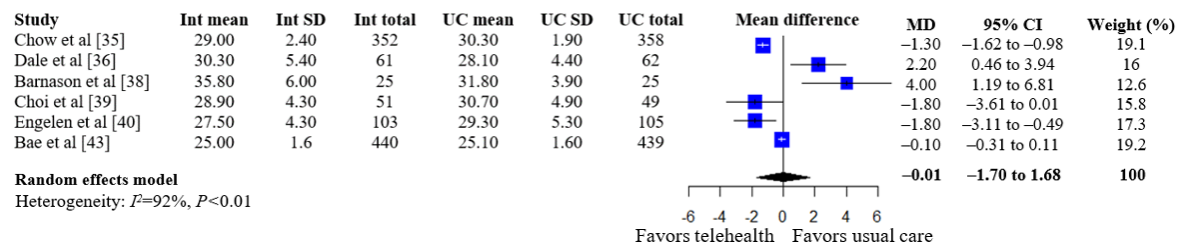
Forest plot of standardized mean differences in body mass index for telehealth-based dietary intervention (Int) and usual care (UC) groups [35,36,38-40,43].

**Figure 4:**
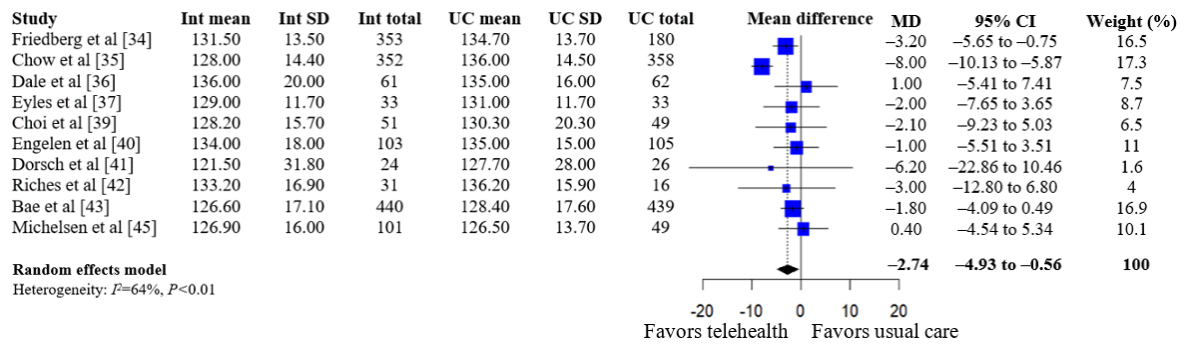
Forest plot of mean differences in systolic blood pressure for telehealth-based dietary intervention (Int) and usual care (UC) groups [34-37,39-43,45].

**Figure 5:**
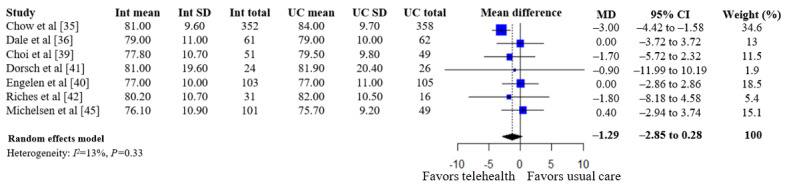
Forest plot of mean differences in diastolic blood pressure levels for telehealth-based dietary intervention (Int) and usual care (UC) groups [35,36,39-42,45].

**Figure 6:**
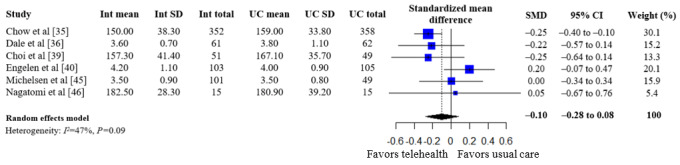
Forest plot of standardized mean differences in total cholesterol levels for telehealth-based dietary intervention (Int) and usual care (UC) groups [35,36,39,40,45,46].

**Figure 7:**
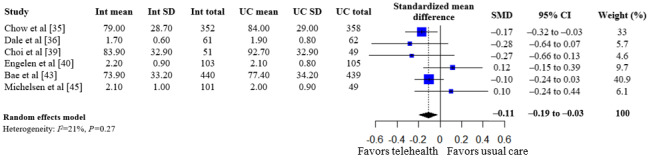
Forest plot of standardized mean differences in low-density lipoprotein cholesterol levels for telehealth-based dietary intervention (Int) and usual care (UC) groups [35,36,39,40,43,45].

**Figure 8:**
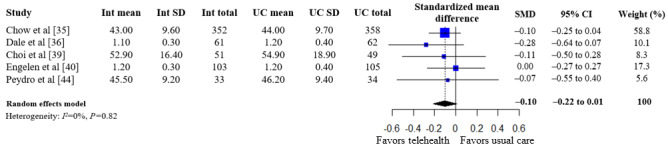
Forest plot of standardized mean differences in high-density lipoprotein cholesterol levels for telehealth-based dietary intervention (Int) and usual care (UC) groups [35,36,39,40,44].

**Figure 9:**
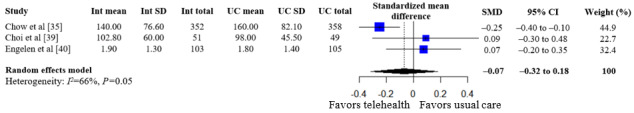
Forest plot of standardized mean differences in triglyceride levels for telehealth-based dietary intervention (Int) and usual care (UC) groups [35,39,40].

**Figure 10:**
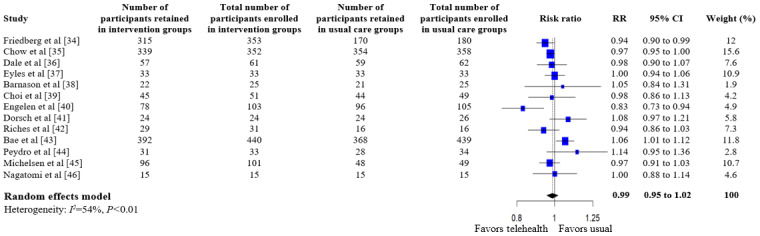
Forest plot of risk ratio (RR) for feasibility [34-46].

A summary of the pooled estimates for all outcomes is reported in Table 2. Telehealth-based dietary interventions significantly reduced systolic blood pressure (MD –2.74, 95% CI –4.93 to –0.56) and LDL cholesterol (SMD –0.11, 95% CI –0.19 to –0.03) when compared to UC among individuals with CVD. The pooled estimates for weight (SMD –0.15, 95% CI –0.34 to 0.04), BMI (MD –0.01, 95% CI –1.70 to 1.68), diastolic blood pressure (MD –1.29, 95% CI –2.85 to 0.28), and levels for total cholesterol (SMD –0.10, 95% CI –0.28 to 0.08), HDL (SMD –0.10, 95% CI –0.22 to 0.01), and triglycerides (SMD –0.07, 95% CI –0.32 to 0.18) showed no significant difference among individuals with CVD who received telehealth-based dietary interventions versus UC. The forest plots and pooled estimate results for individual clinical outcomes are presented in Figures 2-9. The feasibility of intervention groups of the included RCTs did not significantly differ from UC groups (risk ratio 0.99, 95% CI 0.95 to 1.02), as shown in Figure 10, indicating similar feasibility between the 2 groups.

**Table 2:** Summary of meta-analysis results.

Outcomes	Number of studies (n=13), n (%)	Participants, n	Statistical methods	Effect estimates (95% CI)	Heterogeneity, *I*^2^ (%)^a^ (*P* value)
Weight	4 (31)	425	SMD^b^ (IV^c^, random, 95% CI)	–0.15 (–0.34 to 0.04)	0 (.49)
BMI	6 (46)	2070	MD^d^ (IV, random, 95% CI)	–0.01 (–1.70 to 1.68)	92 (<.01)
Systolic blood pressure	10 (77)	2866	MD (IV, random, 95% CI)	–2.74 (–4.93 to –0.56)	64 (<.01)
Diastolic blood pressure	7 (54)	1388	MD (IV, random, 95% CI)	–1.29 (–2.85 to 0.28)	13 (.33)
Total cholesterol	6 (46)	1321	SMD (IV, random, 95% CI)	–0.10 (–0.28 to 0.08)	47 (.09)
LDL^e^ cholesterol	6 (46)	2170	SMD (IV, random, 95% CI)	–0.11 (–0.19 to –0.03)	21 (.27)
HDL^f^ cholesterol	5 (38)	1208	SMD (IV, random, 95% CI)	–0.10 (–0.22 to 0.01)	0 (.82)
Triglycerides	3 (23)	1018	SMD (IV, random, 95% CI)	–0.07 (–0.32 to 0.18)	66 (.05)
Feasibility	13 (100)	3013	RR^g^ (M-H^h^, random, 95% CI)	0.99 (0.95 to 1.02)	54 (<.01)

^a^Values for n not provided for *I*^2^ percentages.

^b^SMD: standardized mean difference.

^c^IV: inverse-variance model.

^d^MD: mean difference.

^e^LDL: low-density lipoprotein.

^f^HDL: high-density lipoprotein.

^g^RR: risk ratio.

^h^M-H: Mantel-Haenszel model.

Funnel plot and the Egger statistic were assessed only for the feasibility outcome because its analysis included more than 10 RCTs, which ensured adequate power to test for publication bias [29]. Visual symmetry was noted in the funnel plot for the outcome, indicating no significant evidence for publication bias, as shown in Multimedia Appendix 3. This result was confirmed by the 1-tailed Egger regression test estimate for the feasibility outcome (t_11_=0.51, *P*=.62).

### Sensitivity Analyses

Sensitivity analyses were conducted for the systolic blood pressure and the feasibility (ie, feasibility) outcomes to investigate the significant heterogeneity detected. These outcomes were deemed appropriate for further assessment because they involved more than 7 RCTs, ensuring that the *I*^2^ estimate was reliable and unbiased by the small number of studies analyzed [30]. The results for the sensitivity analyses on intervention type and follow-up duration after intervention initiation are shown in Multimedia Appendix 4 [34-37,39-43,45], Multimedia Appendix 5 [34-37,39-43,45], Multimedia Appendix 6 [34-46], and Multimedia Appendix 7 [34-46]. App-based (MD –1.33, 95% CI –4.41 to 1.74) and text-based (MD –3.44, 95% CI –8.61 to 1.72) interventions did not show any significant difference between telehealth-based dietary interventions and UC for systolic blood pressure (Multimedia Appendix 4 [34-37,39-43,45]). The RCTs that involved app-based interventions were nonsignificantly homogeneous (*I*^2^=0%, *P*=.91); however, RCTs with text-based interventions had significant and considerable heterogeneity (*I*^2^=89%, *P*<.01; Multimedia Appendix 4 [34-37,39-43,45]). The RCTs with a 24-week follow-up duration after intervention initiation showed a significant reduction in systolic blood pressure when compared to UC (MD –3.53, 95% CI –7.05 to –0.01) but they had significant and considerable heterogeneity (*I*^2^=85%, *P*<.01; Multimedia Appendix 5 [34-37,39-43,45]).

The sensitivity analyses for the feasibility outcome consistently showed no significant difference between telehealth-based dietary interventions and UC groups (Multimedia Appendices 6 [34-46] and 7 [34-46]). The RCTs involving app-based interventions had insignificant and low heterogeneity (*I*^2^=14%, *P*=.33), while RCTs with text-based interventions demonstrated significant and considerable heterogeneity (*I*^2^=78%, *P*=.01; Multimedia Appendix 6 [34-46]). Within the feasibility outcome, RCTs with a 12-week follow-up duration after intervention initiation were insignificantly homogeneous (*I*^2^=0%, *P*=.86), while RCTs with a 24-week follow-up duration showed significant and considerable heterogeneity (*I*^2^=74%, *P*<.01; Multimedia Appendix 7 [34-46]).

### Influence Analysis

An influence analysis was conducted to determine whether any particular RCT impacted the heterogeneity for the systolic blood pressure and the feasibility outcomes, as shown in Multimedia Appendices 8 [34-37,39-43,45] and 9 [34-46]. The results for the systolic blood pressure outcome confirmed that the RCT by Chow et al [35] was the main source of heterogeneity. After omitting this RCT from the analysis, the MD summary estimate still showed telehealth-based dietary interventions to reduce systolic blood pressure significantly more than UC (MD –1.94, 95% CI –3.31 to –0.58); however, the *I*^2^ percent of this outcome dropped from 64.4% (substantial heterogeneity) to 0% (low heterogeneity; Multimedia Appendix 8 [34-37,39-43,45]).

For the feasibility outcome, no single RCT was determined to be drastically different from the others in that its omission from analysis would reduce the outcome’s moderate heterogeneity to the low category (Multimedia Appendix 9 [34-46]).

## Discussion

### Principal Findings

To the best of our knowledge, this study is the first to assess the effectiveness and feasibility of telehealth-based dietary interventions among individuals with CVD. Our study showed that telehealth-based dietary interventions improved levels of systolic blood pressure and LDL cholesterol when compared to UC. We further found no significant difference between telehealth-based dietary interventions and UC in improving weight, BMI, levels of diastolic blood pressure, total and HDL cholesterol, and triglycerides among individuals with CVD. There was no significant difference in feasibility between telehealth-based dietary interventions and UC.

Our finding of a positive clinical effect of telehealth-based dietary interventions on LDL cholesterol levels was in contrast to a previous meta-analysis conducted by Kelly et al [47] that found no significant differences in LDL cholesterol when comparing the effectiveness of telehealth-based dietary interventions to UC in changing dietary habits among chronic disease patients. However, our results of telehealth interventions improving systolic blood pressure compared to UC were confirmed by a meta-analysis by Kelly et al [47]. Plausible explanations for the clinical effectiveness of telehealth-based dietary interventions observed in our study are rooted in the unique benefits that telehealth-based technologies provide in modifying health behaviors. Telephone-based dietary telehealth interventions likely motivated participants through easily accessible, monthly scheduled, individually tailored counseling sessions, aimed to improve multiple components of their diet (ie, the intake of sodium, fat, fruit, and vegetables) [34]. According to Bandura’s social cognitive theory (SCT), a motivated attitude can lead an individual to carry out healthy behaviors [48]. Telehealth-based dietary interventions that involved text messages were significantly more effective than UC in improving systolic blood pressure levels, possibly as a result of increasing and reinforcing participants’ knowledge of healthy behaviors through receipt of regular messages, which may impact an individual’s self-efficacy, and thereby leading to healthy behaviors per the SCT [36,48]. An RCT by Dale et al [36], which was included in our analysis, aimed to determine the effectiveness of a text message-based cardiac rehabilitation intervention among adults with coronary heart disease. Through the intervention in this trial, participants received recommendations to improve lifestyle behaviors, including diet [36]. Adherence to the recommendations and clinical biomarkers were measured as the primary and secondary outcomes, respectively [36]. The number of text messages sent to participants in the RCT’s intervention arm was reduced from the first 12 weeks (7 messages per week) to the last 12 weeks (5 messages per week) of the study period [36]. As the number of text messages from the study team decreased per week, participants may have relapsed into unhealthy dietary behaviors [36]. This suggests a need for sustained implementation of telehealth-based dietary interventions as a continued source of knowledge and support of self-efficacy for participants’ healthy behaviors and their clinical benefits. However, future research should confirm whether effectiveness is sustained if intervention durations are prolonged. Furthermore, findings from an RCT included in our study assessed the effectiveness and feasibility of a telehealth-based weight management intervention with cardiac rehabilitation in reducing the weight of overweight and obese individuals with CVD postcardiac procedure when compared to those who only received cardiac rehabilitation [38]. This RCT showed that participants who received nutritional intervention through an audio-visual media device in addition to cardiac rehabilitation had higher scores of perceived self-efficacy, knowledge, and skills in CVD self-management during follow-up than those who only received cardiac rehabilitation [38]. Greater self-efficacy, knowledge, and skill are all known constructs of the SCT, shown to help participants improve their health behaviors [48]. Lastly, a telehealth-based dietary intervention that involved smartphone apps provided notifications on heart-healthy alternatives to participants at the time of eating in a restaurant or purchasing food at a grocery store [41]. Such real-time app notifications provided participants with tailored nutritional information during critical decision-making time, which may be a key to the clinical benefits of telehealth-based dietary interventions observed in our study [41]. Various types of app-based interventions have been implemented through telehealth in the included RCTs that engage participants in different frequencies and timings during the day and target different constructs of behaviors, all aiming to improve cardiovascular health outcomes. For example, some app-based telehealth interventions may provide clinical benefits among individuals with CVD because they require participants to enter their food consumption into the app daily, which may improve motivation toward adhering to a heart-healthy diet [44,48]. According to the SCT, improved motivation can increase skills toward health behaviors by increasing participants’ capacity for forethought and goal setting [48].

While all included RCTs provided a nutritional component to participants (UC and telehealth intervention groups), the benefits of the telehealth-based dietary intervention over UC may imply the need for improved access to and standard of nutritional care for individuals diagnosed with CVD [34-46]. Participants enrolled in usual cardiac rehabilitation postcardiac events and procedures were provided with weekly educational sessions for at least 2 weeks, and nutrition was only 1 out of several other topics covered [36,38,44,45]. Once cardiac rehabilitation was complete, participants were typically not followed up by the rehabilitation unit, which indicates that nutrition was likely a very small component of the overall rehabilitation care given to the participants [44]. Other forms of UC involved regular outpatient clinic visits where participants received disease-specific pharmacological treatment, evaluation of cardiovascular risk factors, and general feedback on improving their lifestyle habits [35,37,40,43,46]. In some of the included RCTs, UC simply implied that participants received nutritional and disease-specific advice at 1 time point only (baseline) [34]. In one of the included RCTs, participants in the UC group received only a postal copy of publicly available booklets from the British Heart Foundation focused on reducing salt intake, titled “Cut Down on Salt” or “Taking Control of Salt” [42]. Apart from 1 RCT, where an RDN provided nutritional counseling to participants in the UC group [39], nutritionally trained health care professionals may not have been consistently involved in the dissemination of nutritional care in UC groups of the included RCTs.

While most included RCTs did not clearly state that they involved RDNs to disseminate the nutritional component to participants in intervention and UC groups, there is strong evidence suggesting that patients referred to RDNs have significant improvements in their clinical CVD risk factor outcomes [49-52]. According to the Academy of Nutrition and Dietetics, RDNs are increasingly using telehealth services in their clinical practice [53]. A qualitative study of 200 RDNs reported that medical nutrition therapy delivered through telehealth services positively impacted patient outcomes [54]. Previous studies suggest that telehealth services enable RDNs to better understand their patients’ nutritional habits since they were able to observe and discuss patients’ home environment more efficiently than in the clinical setting [54,55]. Furthermore, telehealth services may allow RDNs to facilitate group visits with a patient to involve their family members more conveniently during dietary education sessions, which has been shown to improve patient adherence to dietary advice and attendance to medical nutrition therapy appointments with RDNs [54,55].

Despite the benefits of telehealth-based dietary interventions on clinical CVD risk factors, some policy and patient-based barriers may prevent the implementation of telehealth use in clinical practice. First, each state has its own policies and license requirements for telehealth-based dietary practices in the clinical setting [53]. This may create inconsistencies in nutritional care provided to individuals with CVD nationwide. Therefore, more research showing the clinical benefits of telehealth-based dietary interventions is needed to demonstrate their effectiveness to policymakers in an attempt to standardize policies and strengthen dietetics practices across the United States [53]. Additionally, RDNs may not be able to use telehealth services to communicate with specific patients who have a limited understanding of communication technologies [53]. This patient-related barrier must be considered by RDNs on a case-by-case basis before engaging in virtual care.

### Limitations

Our study is not without limitations. First, the included RCTs did not consistently provide participant demographic information on race, and levels of education and income, which limits our understanding of participant characteristics and the potential impact they may have on the overall results. Next, a majority of the included RCTs were of moderate overall quality, which may have impacted the overall results of our study. While we did not assess the certainty of evidence through the GRADE (Grading of Recommendations, Assessment, Development, and Evaluations) approach, we were able to assess the quality of each RCT using the Cochrane risk of bias tool, analyze publication bias when appropriate, and evaluate heterogeneity in the meta-analysis results to determine any source of bias. The included RCTs did not provide adequate data to analyze the effectiveness of telehealth-based dietary intervention on improving blood glucose levels or hemoglobin A_1C_. While one of the assessed outcomes involved 13 RCTs, most other clinical CVD risk factor outcomes only involved 3-7 RCTs, which limited our analysis of heterogeneity and publication bias. Additionally, our sensitivity analysis was limited by the few number of RCTs available for each type of telehealth-based dietary intervention and the length of follow-up duration after intervention initiation. Therefore, more RCTs are needed to build a stronger evidence base on the effectiveness of different types of telehealth-based dietary interventions and for various follow-up durations to confirm our results. While we were able to determine that the RCT by Chow et al [35] was the source of heterogeneity for the systolic blood pressure outcome, this was less clear for the feasibility outcome. Nonetheless, our study contributes valuable knowledge on the effectiveness and feasibility of telehealth-based dietary interventions among individuals with CVD using robust meta-analysis techniques.

### Conclusions

Telehealth-based dietary interventions significantly improved systolic blood pressure and LDL cholesterol when compared to UC among individuals with CVD. Future updates on this meta-analysis are needed to evaluate data from an increased number of RCTs for each type of telehealth intervention and follow-up duration in individuals with CVD.

## Appendix

Multimedia Appendix 1 Search strategy.

Multimedia Appendix 2 Characteristics of the included randomized controlled trials.

Multimedia Appendix 3 Funnel plot of risk ratio for feasibility.

Multimedia Appendix 4 Forest plot of mean differences in systolic blood pressure levels for telehealth-based dietary intervention (Int) and usual care (UC) groups by intervention type.

Multimedia Appendix 5 Forest plot of mean differences in systolic blood pressure levels for telehealth-based dietary intervention (Int) and usual care (UC) groups by follow-up duration after intervention initiation.

Multimedia Appendix 6 Forest plot of risk ratio (RR) for feasibility by intervention type.

Multimedia Appendix 7 Forest plot of risk ratio (RR) for feasibility by follow-up duration after intervention initiation.

Multimedia Appendix 8 Influence analysis of mean differences (MD) for systolic blood pressure levels.

Multimedia Appendix 9 Influence analysis of risk ratio (RR) for feasibility.

Multimedia Appendix 10 PRISMA 2020 checklist.

## Abbreviations

CVD: cardiovascular disease
GRADE: Grading of Recommendations, Assessment, Development, and Evaluations
HDL: high-density lipoprotein
LDL: low-density lipoprotein
MD: mean difference
PRISMA: Preferred Reporting Items for Systematic Reviews and Meta-Analyses
RCT: randomized controlled trial
RDN: registered dietitian nutritionist
SCT: social cognitive theory
SMD: standardized mean difference
UC: usual care

## Data Availability

All data generated or analyzed during this study are included in the tables and figures of this published paper.
