# Supplementary material for: Effectiveness and Feasibility of Telehealth-Based Dietary Interventions Targeting Cardiovascular Disease Risk Factors: Systematic Review and Meta-Analysis
Source: J Med Internet Res. 2024 Feb 16;26:e49178. doi: 10.2196/49178 (PMC10907949; doi:10.2196/49178)
Supplement: Multimedia Appendix 1 [file jmir_v26i1e49178_app1.pdf]

PubMed Search String:

(“diet” or “nutrition” or “nutrition status” or “status, nutrition” or “nutritional science” or “science, nutritional” or “nutrition science” or “science, nutrition”) and (“telehealth” or “telemedicine” or “mobile health” or “health, mobile” or “mhealth” or “telehealth” or “ehealth”) and ("cardiovascular disease" or “cardiovascular diseases” or “disease, cardiovascular” or “diseases, cardiovascular” or “heart disease” or “cardiac disease” or “cardiac disorder” or “heart disorder” or “vascular disease” or “disease, vascular”) and (“rand*”).

Search in July, 2022
No Filters used

Cochrane Library Search String:

(“diet” or “nutrition” or “nutrition status” or “status, nutrition” or “nutritional science” or “science, nutritional” or “nutrition science” or “science, nutrition”) and (“telehealth” or “telemedicine” or “mobile health” or “health, mobile” or “mhealth” or “telehealth” or “ehealth”) and ("cardiovascular disease" or “cardiovascular diseases” or “disease, cardiovascular” or “diseases, cardiovascular” or “heart disease” or “cardiac disease” or “cardiac disorder” or “heart disorder” or “vascular disease” or “disease, vascular”) and (“rand*”).

Search in July, 2022
No Filters used

Web of Science Search String:

(“diet” or “nutrition” or “nutrition status” or “status, nutrition” or “nutritional science” or “science, nutritional” or “nutrition science” or “science, nutrition”) and (“telehealth” or “telemedicine” or “mobile health” or “health, mobile” or “mhealth” or “telehealth” or “ehealth”) and ("cardiovascular disease" or “cardiovascular diseases” or “disease, cardiovascular” or “diseases, cardiovascular” or “heart disease” or “cardiac disease” or “cardiac disorder” or “heart disorder” or “vascular disease” or “disease, vascular”) and (“rand*”).

Search in July, 2022
No Filters used

ClinicalTrials.gov Search String:

Condition or disease: “cardiovascular diseases”
Other terms: “telehealth”

Search in July, 2022
Filter used: Under the “Study Results” filter, “With Results” option was selected.
