# Supplementary material for: Effectiveness and Feasibility of Telehealth-Based Dietary Interventions Targeting Cardiovascular Disease Risk Factors: Systematic Review and Meta-Analysis
Source: J Med Internet Res. 2024 Feb 16;26:e49178. doi: 10.2196/49178 (PMC10907949; doi:10.2196/49178)
Supplement: Multimedia Appendix 2 [file jmir_v26i1e49178_app2.docx]

**Table S1.** Characteristics of the included randomized controlled trials.

| Study characteristics | | | | | | Participant characteristics | | | |
| --- | --- | --- | --- | --- | --- | --- | --- | --- | --- |
| Author | Trial location; urban or rural setting | ITT^a^ | UC^b^ | Intervention; type | Intervention duration and follow-up after intervention initiation | CVD^c^ condition | Mean age years (SD) | Women, n (%) |  |
|  |  |  |  |  |  |  |  |  |  |
| Friedberg et al [34] | United States; urban | 533 | Nutritional and other advice to manage hypertension | SMI^d^, and HEI^e^; telephonic | 24 weeks and 24 weeks | Hypertension | SMI: 66.4 (8.8); HEI: 66.5 (12.8); UC: 65.4 (10.2) | SMI and HEI: 3 (0.8); UC: 4 (2.2) |  |
| Chow et al [35] | Australia; urban | 710 | Education and counseling on lifestyle change and pharmacotherapy | TextMe; text-based | 24 weeks and 24 weeks | Coronary heart disease | TextMe: 57.9 (9.1); UC: 57.3 (9.3) | TextMe: 65 (18.5); UC: 63 (17.6) |  |
| Dale et al [36] | New Zealand; urban | 123 | Education on lifestyle change, cardiovascular risk factors, and psychosocial support | Text4Heart; text-based | 24 weeks and 24 weeks | Individuals with CVD post cardiac event | Text4Heart: 59.0 (10.5); UC: 59.9 (11.8) | Text4Heart: 13 (21.3);  UC: 10 (16.1) |  |
| Eyles et al [37] | New Zealand; urban | 66 | Education provided on nutrition, exercise, returning to normal activities, mental well-being, and reducing risk of future cardiac events | SaltSwitch; app-based | 4 weeks and 4 weeks | CVD | SaltSwitch: 64.0 (7.0); UC: 65.0 (8.0) | SaltSwitch: 3 (9.1); UC: 8 (24.2) |  |
| Barnason et al [38] | United States; rural | 50 | Education and counseling on managing elevated BMI as a CVD risk factor | WMI^f^; audio-visual media | 12 weeks and 16 weeks | Individuals with CVD post PCI^g^ or CABGS^h^ | Total: 63.0 (9.3) | WMI: 5 (20.0); UC: 8 (32.0) |  |
| Choi et al [39] | United States; urban | 100 | RDN^i^ administered dietary education and counseling | SA EXP^j^, RDN-led intervention; app-based | 12 weeks and 12 weeks | CVD | SA: 57.2 (SE^k^:1.8); UC: 56.6 (SE: 1.7) | SA: 22 (43.1); UC: 17 (34.7) |  |
| Engelen et al [40] | Netherlands; urban | 208 | Evaluation of cardiovascular risk factors and feedback to optimize lifestyle | VV^l^, involved RDN in the health care team; web-based | 48 weeks and 48 weeks | CVD | VV: 63.3 (10.0); UC: 63.7 (9.8) | VV: 30 (29.1); UC: 36 (34.3) |  |
| Dorsch et al [41] | United States; urban | 50 | Usual dietary advice | LowSalt4Life; app-based | 8 weeks and 8 weeks | Hypertension | LowSalt4Life: 56.6 (10.0); UC: 58.2 (11.0) | LowSalt4Life: 14 (58.3); UC: 16 (61.5) |  |
| Riches et al [42] | United Kingdom; urban | 47 | Dietary education provided through one of the British booklets entitled “*Heart Foundation Cut Down on Salt*” or “*Taking Control of Salt*” | SaltSwap; app-based | 6 weeks and 6 weeks | Hypertension | SaltSwap: 64.0 (12.0); UC: 67.0 (7.0) | SaltSwap: 20 (64.5); UC: 10 (62.5) |  |
| Bae et al [43] | Korea; urban | 879 | Medication treatment and education on cardiovascular health and risk factors | TMI^m^, involved RDN in the health care team; text-based | 24 weeks and 24 weeks | Individuals with CVD post PCI | TMI: 60.1 (10.6); UC: 60.7 (10.4) | TMI: 72 (16.4); UC: 75 (17.1) |  |
| Peydró et al [44] | Spain; urban | 67 | Education provided on cardiovascular medication, nutrition, smoking, and exercise | CTR^n^; app-based | 40 weeks and 40 weeks | Acute coronary syndromes | CTR: 57.5 (9.0); UC: 54.7 (9.9) | CTR: 6 (18.2); UC: 7 (20.6) |  |
| Michelsen et al [45] | Sweden; urban | 150 | Lifestyle education and counseling provided to manage risk factors | LifePod; app-based | 25 weeks and 48-56 weeks | Myocardial infarction | LifePod: 60.0 (8.9); UC: 61.1 (8.6) | LifePod: 17 (16.8); UC: 13 (26.5) |  |
| Nagatomi et al [46] | Japan; urban | 30 | Pharmacological and nonpharmacological therapy | ICT^o^, involved RDN in the health care team; app-based | 12 weeks and 12 weeks | Heart failure | ICT: 59.8 (10.0); UC: 67.7 (8.9) | ICT: 6 (40.0); UC: 8 (53.3) |  |
| ^a^ITT: intention-to-treat.  ^b^UC: usual care.  ^c^CVD: cardiovascular disease.  ^d^SMI: stage-matched intervention.  ^e^HEI: health education intervention.  ^f^WMI: weight management intervention.  ^g^PCI: percutaneous coronary intervention.  ^h^CABGS: coronary artery bypass graft surgery.  ^i^RDN: Registered Dietitian Nutritionist.  ^j^SA EXP: smartphone app.  ^k^SE: standard error.  ^l^VV: vascular view.  ^m^TMI: text message intervention.  ^n^CTR: cardiac telerehabilitation.  ^o^ICT: information communication technologies. | | | | | | | | | |
