# Supplementary figures and images for: Effectiveness and Feasibility of Telehealth-Based Dietary Interventions Targeting Cardiovascular Disease Risk Factors: Systematic Review and Meta-Analysis
Source: J Med Internet Res. 2024 Feb 16;26:e49178. doi: 10.2196/49178 (PMC10907949; doi:10.2196/49178)

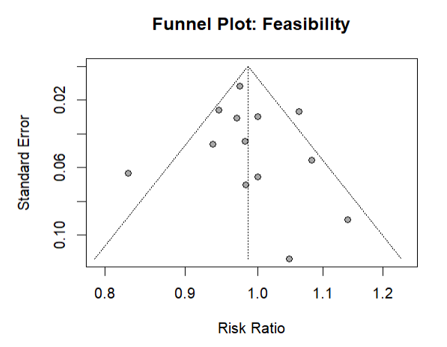

Supplement: Multimedia Appendix 3 [file jmir_v26i1e49178_app3.png]

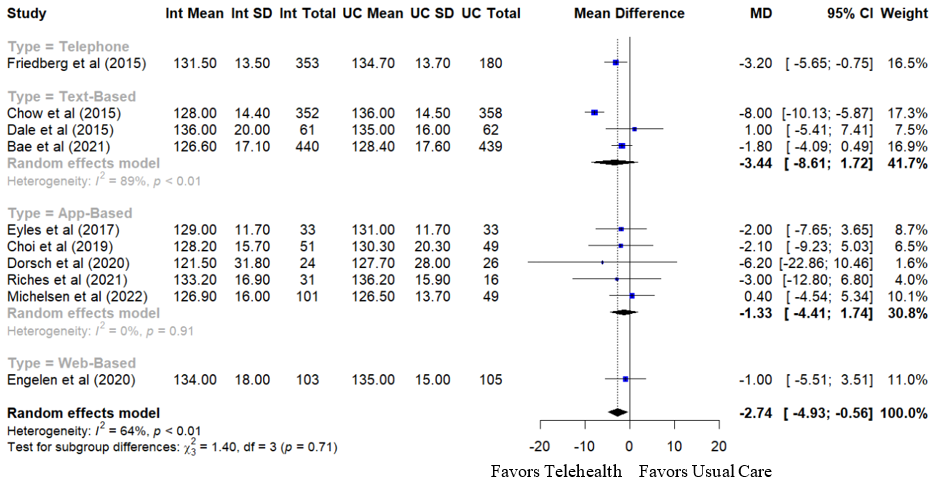

Supplement: Multimedia Appendix 4 [file jmir_v26i1e49178_app4.png]

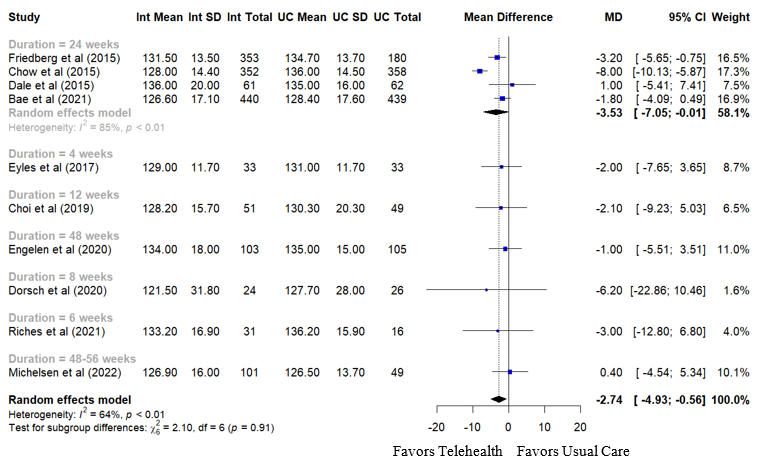

Supplement: Multimedia Appendix 5 [file jmir_v26i1e49178_app5.png]

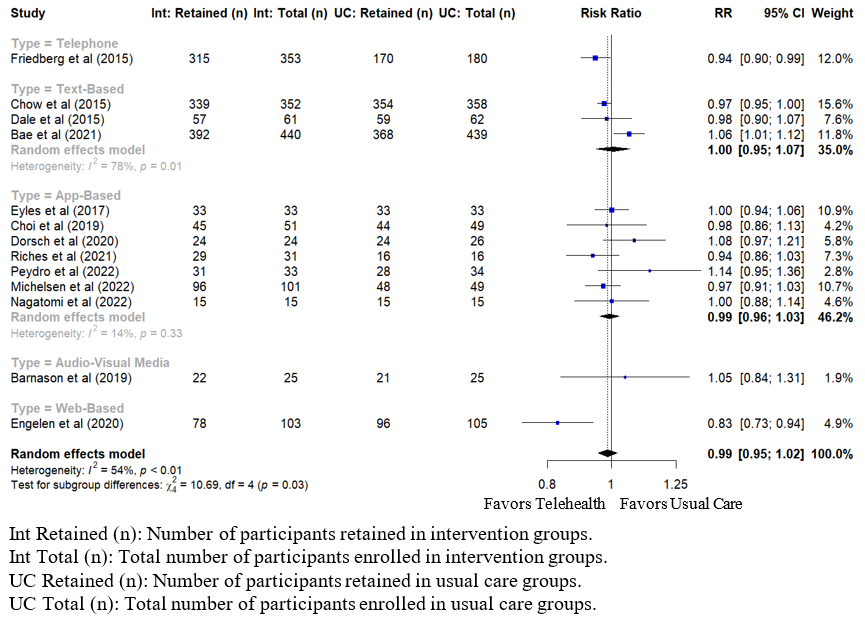

Supplement: Multimedia Appendix 6 [file jmir_v26i1e49178_app6.png]

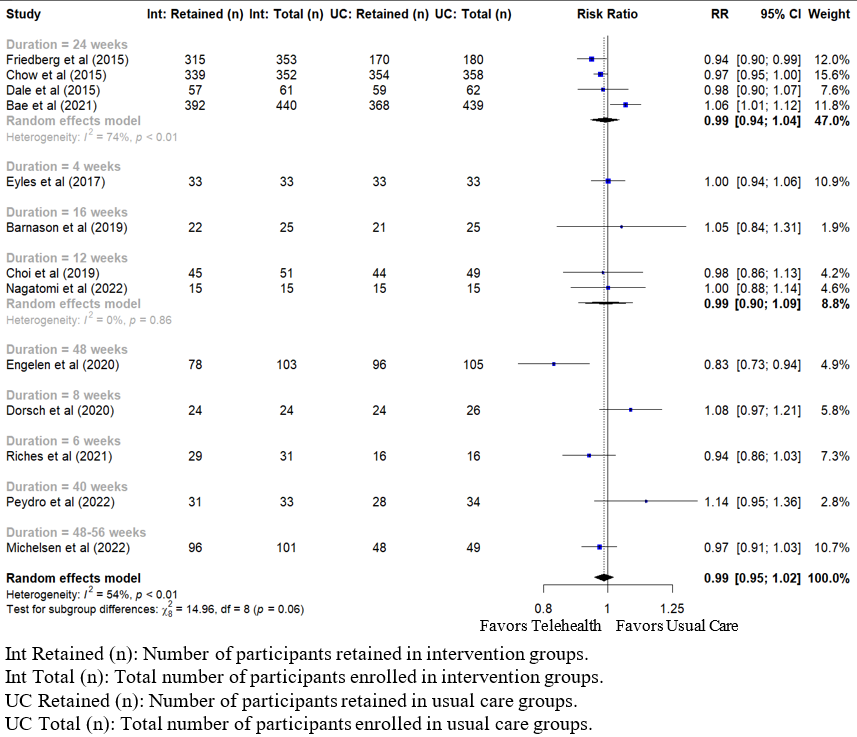

Supplement: Multimedia Appendix 7 [file jmir_v26i1e49178_app7.png]

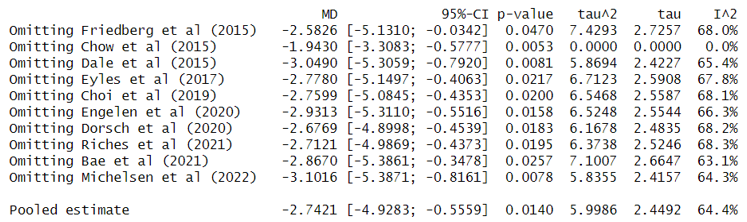

Supplement: Multimedia Appendix 8 [file jmir_v26i1e49178_app8.png]

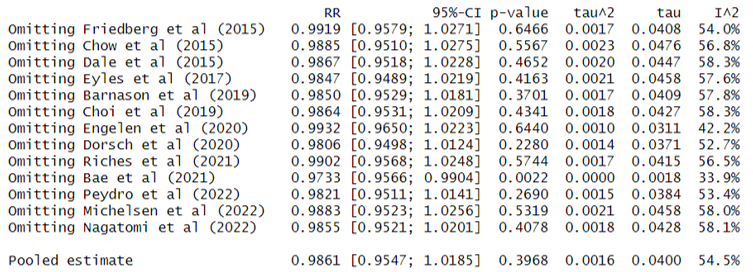

Supplement: Multimedia Appendix 9 [file jmir_v26i1e49178_app9.png]
